# Etiological Roles Are Not Fixed: A Functional–Temporal and Adaptive-Capacity Framework for Painful Temporomandibular Disorders

**DOI:** 10.3390/dj14100684

**Published:** 2026-10-20

**Authors:** Elisa Seiko Wada, Rodrigo Lorenzi Poluha

**Affiliations:** Department of Dentistry, State University of Maringá, Mandacaru Avenue 1550, Maringá 87080-000, PR, Brazil

**Keywords:** painful temporomandibular disorders, orofacial pain, etiology, biopsychosocial model, risk factors, prognosis, effect modification, clinical reasoning

## Abstract

**Background/Objectives:** Painful temporomandibular disorders (TMDs) are multifactorial and biopsychosocial, yet identifying associated factors does not establish the function each factor occupies along an individual clinical trajectory. This conceptual hypothesis article aimed to propose a functional–temporal framework that distinguishes etiological role from biopsychosocial domain and incorporates individual adaptive capacity. **Methods:** The framework was developed from established TMD concepts and purposively selected prospective and longitudinal evidence relevant to first onset, post-onset course, repeated measurement, and person-by-exposure interaction. The article is not a systematic or scoping review. **Results:** Recognized influences are organized into three functional positions: predisposing, initiating, and perpetuating. These positions are not treated as intrinsic properties of variables; assignment depends on temporal ordering, context, measurement, phenotype, and the specified outcome. Adaptive capacity is proposed as a conceptual source of differential response and is distinguished from generic effect modification, which is one empirical strategy for testing the hypothesis. Existing longitudinal studies show that susceptibility, onset-related change, post-onset course, and exposure-by-person interactions can be measured, but no cohort has validated the complete framework. Falsifiable predictions and conservative wording rules are specified. **Conclusions:** The framework offers a temporally disciplined approach to etiological interpretation and patient communication without implying deterministic causation or treatment efficacy. Its current empirical basis is strongest for painful TMD, and densely measured inception cohorts are needed to test whether functional position and prespecified moderators add explanatory or predictive information.

## 1. Introduction

Temporomandibular disorders (TMDs) are a heterogeneous group of musculoskeletal conditions affecting the temporomandibular joints, the masticatory muscles, and associated structures. Contemporary diagnostic frameworks separate pain-related disorders such as myalgia and arthralgia from intra-articular conditions such as disc displacement, degenerative joint disease, and subluxation [1,2]. The distinction matters for etiological reasoning because structural findings and clinically important pain do not always coincide; disc displacement with reduction, for example, is frequently asymptomatic, and the joint tissues often adapt to the altered disc position without pain [3].

For painful TMD, a multifactorial biopsychosocial etiology is well supported [4,5]. The Orofacial Pain: Prospective Evaluation and Risk Assessment (OPPERA) program followed initially TMD-free adults and evaluated sociodemographic, general-health, psychological, clinical orofacial, sensory, autonomic, and genetic domains, showing that first-onset painful TMD arises against a distributed architecture of risk rather than from one dominant determinant [5,6]. Independent cohorts have converged on a similarly broad picture, and a systematic review of cohort studies confirms both the breadth of candidate risk factors and the heterogeneity of the underlying evidence [7].

This body of work identifies influences associated with TMD, but a distinct conceptual problem remains in clinical and etiological reasoning: once several biopsychosocial influences have been recognized in a particular patient or dataset, what functional role can each legitimately be assigned, and at what point in the clinical trajectory? A list of associated variables does not distinguish an influence that has shaped susceptibility for years from one that changed shortly before symptom onset or from one that is associated with persistence after onset. Nor does factor presence alone explain why apparently similar exposure profiles may be followed by different clinical courses.

The novelty of the present proposal does not lie in introducing the terms predisposing, initiating, and perpetuating, which are already established in the TMD literature. Rather, it lies in treating these categories as temporally and contextually assigned functional positions rather than as fixed attributes of specific variables; requiring the assignment to be supported by the temporal structure and measurement of the evidence; and coupling this functional framework with an explicitly testable adaptive-capacity hypothesis based on measurable differential response. Because the prospective evidence available for these questions derives predominantly from painful TMD cohorts, the present framework is centered on painful TMD. Extension to structural or degenerative TMD phenotypes should be tested separately rather than assumed.

### Approach to Evidence Selection

Because this article develops a conceptual hypothesis rather than an evidence synthesis, studies were not selected through a prespecified systematic review protocol. The evidence base was assembled from established conceptual sources and the prospective literature central to TMD etiological research, particularly the OPPERA program, and was supplemented by longitudinal studies relevant to first onset, persistence or recovery, measurement, and person-by-exposure interaction. Priority was given to studies whose design permitted temporal ordering, repeated measurement, post-onset follow-up, or effect modification to be examined. The resulting selection is illustrative rather than exhaustive; no duplicate screening, formal risk-of-bias assessment, or certainty grading was undertaken.

## 2. Frameworks That Inform the Proposal

Four bodies of work supply the components of the framework proposed here, and Table 1 summarizes how each relates to it.

### 2.1. The Biopsychosocial Account

The biopsychosocial perspective established that painful TMD cannot be reduced to a local mechanical lesion. Biological, psychological, and social vulnerabilities interact with contextual and environmental influences, and contemporary reviews accordingly describe TMD etiology as multifactorial [4]. In the present framework, these domains are preserved intact. The three functional positions are not alternative domains but a second axis: a psychological influence remains psychological in nature while occupying whichever functional position its temporal relationship with the specified outcome supports.

### 2.2. Prospective Multidomain Evidence

OPPERA provides the principal prospective foundation for painful TMD by examining many putative risk domains in the same cohort. Multivariable modeling of phenotypic risk factors showed that incidence is predicted by variables drawn from several domains simultaneously, with modest individual effects [6], and the program’s synthesis emphasizes a distributed architecture involving psychological characteristics, pain amplification, general health, clinical variables, and genetic influences [5,8]. This evidence base permits influences to be located in time because measurements occurred before onset, around onset, and afterward.

### 2.3. The Predisposing–Initiating–Perpetuating Vocabulary

The distinction between factors that increase susceptibility, factors associated with onset, and factors that interfere with resolution or promote progression is long established in the TMD literature, which has also noted that a single factor may serve one or several of these roles [4,9]. The vocabulary is useful because it poses three different questions rather than one. The contribution proposed here is therefore not terminological. It is a rule of assignment: a factor should receive a predisposing, initiating, or perpetuating label only when its timing, measurement, and relationship to a specified outcome support that function, rather than because the factor is conventionally associated with one category.

### 2.4. Adaptation as a Counterweight to Disruption

Parker described a dynamic model in which disruptive influences on the masticatory system are balanced against factors that return it toward homeostasis [10]. The underlying conceptual observation is that comparable disturbances may produce different consequences in different individuals. We do not equate Parker’s homeostatic model with statistical effect modification, nor do we suggest that the original model anticipated this analytical formulation. Rather, the qualitative idea of heterogeneous response provides a starting point for a testable proposition: the association between an etiological influence and a clinical outcome may vary according to independently measured person-level characteristics. Effect-modification analysis is one empirical route for evaluating that proposition.

**Table 1:** Established frameworks and their relationship to the proposed functional–temporal framework.

Framework	Principal Contribution	Dimension Emphasized	Relation to the Functional–Temporal Framework
Biopsychosocial account [4]	Situates biological, psychological and social influences within one multifactorial understanding and displaces single-cause explanations.	Which domains of influence are relevant.	Supplies the domains from which predisposing, initiating, and perpetuating influences, and adaptive moderators, may arise. Domain and function are separate axes.
OPPERA prospective cohort [5,6]	Prospective multidomain evaluation of initially TMD-free adults, showing that risk of first-onset painful TMD is distributed across domains with modest individual effects.	How risk is distributed and how it is measured before onset.	Provides evidence in which influences are located in time, allowing functional positions to be examined empirically.
Predisposing–initiating–perpetuating vocabulary [4,9]	Distinguishes susceptibility, onset and interference with resolution, and notes that a single factor may serve one or several of these roles [9].	When and how an influence acts.	Provides the organizing vocabulary; functional assignment is made conditional on temporal and contextual evidence rather than on factor identity.
Dynamic model of adaptation [10]	Balances disruptive influences against factors that return the masticatory system toward homeostasis.	Why comparable demands have different consequences in different people.	Motivates the adaptive dimension; the present framework translates heterogeneous response into a testable proposition that may be examined through prespecified moderation or effect-modification analyses.

## 3. The Functional–Temporal Framework and Its Testable Hypotheses

Within the multifactorial biopsychosocial understanding of painful TMD, we distinguish the organizing framework from the hypotheses it generates. The framework defines three functional positions along a clinical trajectory—predisposing, initiating, and perpetuating. Two related hypotheses are then proposed: first, that functional roles are temporally and contextually assigned rather than intrinsic to variables; and second, that measurable person-level characteristics may modify the impact of etiological influences. This distinction separates a clinical classification structure from claims that can be evaluated empirically.

### 3.1. The Framework: Three Functional Positions

The predisposing position concerns susceptibility. An influence occupying it is present before the clinical episode and alters the conditions under which later perturbations produce clinically relevant symptoms. It may come from any domain—genetic, sensory, systemic, psychological, behavioral, anatomical, social or environmental—and predisposition is not disease: it shifts readiness, not diagnosis.

The initiating position concerns the transition into clinical expression. It may be occupied by a discrete event, by an escalation in a time-varying state, or by another perturbation standing in a plausible temporal relation to onset. It need not be mechanical. Mechanical injury is the most intuitive example, but a physiological, behavioral, psychosocial or systemic change may occupy the same position when the temporal evidence supports it.

The perpetuating position concerns what happens after onset: processes that sustain the disturbance and thereby influence duration, recurrence or the pace of remission. It is not a synonym for chronicity, and it is not assumed to be strongly occupied in every episode.

Two clarifications keep the framework bounded. First, three positions do not require three factors. One influence may occupy two or all three positions in a given trajectory, and several influences may jointly occupy one. Second, the three positions define distinct questions that a complete account of a trajectory may ask; they do not guarantee that every question has a substantial answer in every episode. A brief, self-limited episode may resolve without any maintenance process that is clinically distinguishable, in which case the perpetuating position may be weakly occupied or effectively empty. Whether clinically verified persistent episodes routinely contain an identifiable post-onset maintenance process remains an empirical question rather than a definitional assumption.

### 3.2. Hypothesis 1: Functional Roles Are Temporally and Contextually Assigned

The framework becomes empirically substantive only if functional assignment depends on temporal and contextual evidence. Predisposing, initiating, and perpetuating describe what an influence is doing in relation to a specified outcome, not what the influence intrinsically is. The same variable may occupy different positions depending on when it is present, whether it changes, which outcome is being studied, how it was measured, which other influences are present, and which individual is exposed. Baseline sleep disturbance that predicts later incidence supports a predisposing assignment, but it does not become an initiating influence unless a temporally resolved change or exposure near onset is demonstrated. A psychosocial influence may represent long-standing susceptibility in one patient, an onset-related escalation in a second, and a maintenance influence in a third.

The testable principle is therefore that temporal position, measurement, and outcome should alter the functional inference available for a variable. If well-measured variables consistently retain the same functional role regardless of timing or outcome, the role-plasticity component of the framework would be weakened.

### 3.3. Hypothesis 2: Adaptive Capacity May Modify the Impact of Etiological Influences

Adaptive capacity denotes the conceptual proposition that independently measurable person-level characteristics or processes may alter the magnitude or direction of response to etiological influences across the clinical trajectory. Two individuals may share apparently similar predisposing, initiating, and perpetuating influences and still differ in clinical expression or course because the impact of those influences may differ between them.

Adaptive capacity is not synonymous with generic effect modification. Effect modification is a statistical property of an exposure–outcome association: the magnitude or direction of that association differs across levels of another variable. Adaptive capacity is the conceptual claim that some independently measurable person-level characteristics may systematically contribute to such differential response. Effect-modification analysis is therefore one empirical strategy for testing the adaptive-capacity hypothesis, not its definition. Not every statistical interaction should be interpreted as adaptive capacity; such an interpretation requires an a priori rationale, independent measurement of the proposed moderator, temporal plausibility, and preferably replication. The concept must also resist circularity: stating that a patient remained asymptomatic because adaptation was good explains nothing if adaptation is inferred from the outcome itself. Candidate moderators may be biological, sensory, psychological, behavioral, or contextual, and the same variable may play different analytical roles in different studies. Adaptive capacity may also change over time through healing, learning, coping, altered pain modulation, affective change, or worsening comorbidity. No single latent resilience trait is assumed; the empirical prediction is measurable differential response between and within individuals.

**Figure 1:**
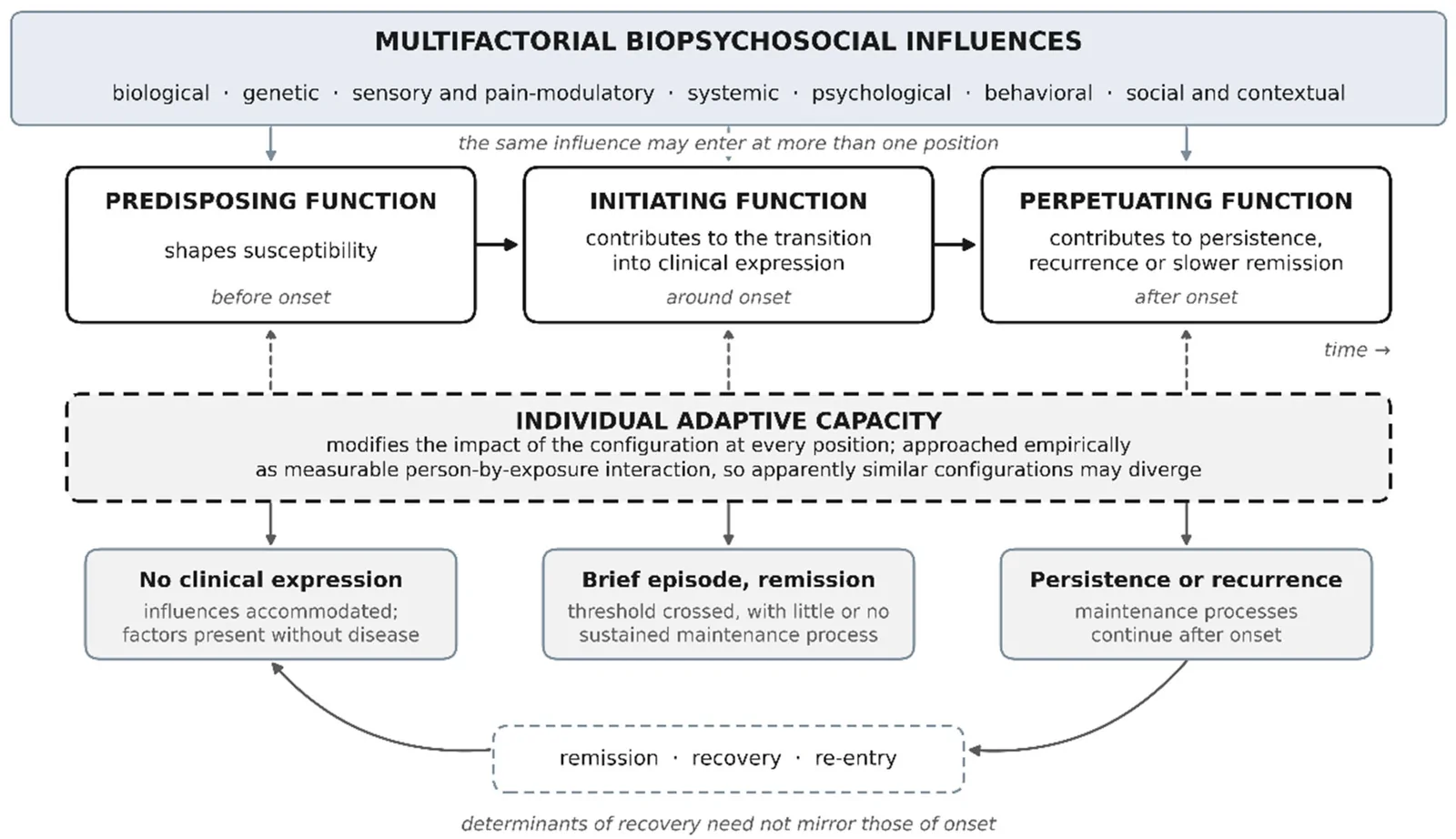
Conceptual representation of the functional–temporal and adaptive-capacity framework. Influences from any biopsychosocial domain may occupy a predisposing, initiating, or perpetuating position along an individual trajectory, and the same influence may occupy more than one position. Individual adaptive capacity is proposed to modify the impact of etiological influences at each position, so that apparently similar configurations may end in no clinical expression, a brief episode with remission, or persistence and recurrence; determinants of recovery need not mirror those of onset. The arrows represent a reasoning sequence, not a deterministic biological pathway.

The relationship among the three functional positions, role plasticity, and the adaptive-capacity hypothesis is summarized in Figure 1.

## 4. Longitudinal Evidence Interpreted Through the Framework

The studies below are not offered as validation of the framework, nor as a reinterpretation of their authors’ conclusions. They show that susceptibility, onset-related exposures, post-onset course, and person-by-exposure interaction can be measured within the existing evidence base, and they illustrate the evidentiary discipline required by the framework. No cohort was designed to test the full formulation.

### 4.1. Susceptibility Is Distributed Across Domains

In OPPERA, psychological measures, general health status, clinical orofacial characteristics, and experimental pain responses each showed prospective associations of varying magnitude with first onset [11,12,13,14], with sleep disturbance and smoking emerging as independent predictors alongside comorbid pain conditions [13]. Independent cohorts report prospective associations involving somatic symptoms, other pain conditions, and depressive and anxiety symptoms, the latter in a population-based cohort using clinical palpation to define TMD pain [15,16]. These findings support predisposition as a distributed function rather than a single lesion, but they do not establish that every baseline predictor is causal or that a predictor retains the same role once the disorder has begun.

### 4.2. The Initiating Position Depends on Temporal Resolution

Initiation is the position for which measurement timing is decisive. In OPPERA, jaw injury was ascertained through quarterly follow-up questionnaires and modeled as a time-dependent covariate; incident injury was strongly associated with subsequent incident painful TMD, and most reported injuries were intrinsic—attributed to sustained mouth opening or yawning rather than to external trauma [17]. The evidential value lies in temporal ordering: a dated exposure that precedes a subsequently classified onset can support an initiating assignment in a way that a cross-sectional association between injury history and prevalent TMD cannot.

Time-varying psychosocial measurement provides a second example. Perceived stress assessed repeatedly before incident TMD increased during the pre-onset period, and the association with incidence differed by COMT diplotype [18]. This does not mean that stress is an initiating influence in every patient. It shows that a changing psychosocial state can be studied with sufficient temporal resolution to permit an initiating interpretation in some trajectories.

### 4.3. The Post-Onset Landscape Is Not the Pre-Onset Landscape Continued

Once painful TMD is established, the prognostic picture changes. Among people with examiner-classified first-onset TMD, clinical measures assessed at onset discriminated persistent from transient cases better than psychosocial measures did, with greater baseline pain intensity and more physical symptoms the main Axis II contributors [19]; in patients with established TMD pain, catastrophizing and depression predicted progression of pain and disability over 18 months [20], and a recent systematic review of prognostic factors for pain persistence and chronification reports a correspondingly biopsychosocial but heterogeneous set of predictors [21]. OPPERA analyses that re-measured premorbid risk factors at onset and six months later found that measures of change improved prediction of incidence beyond premorbid measurement alone [22].

Recovery is likewise not simply risk-reversed. Long-term OPPERA data show biopsychosocial characteristics moving in different directions across people who develop, retain or remit from TMD, with several parameters improving in participants whose pain persisted for years—an observation the authors interpret as evidence of ongoing coping and adaptation [23]. In a general-population cohort screened repeatedly for TMD symptoms, transition rates between symptom states differed by sex, with women showing both a higher rate of transition into symptoms and a lower rate of transition out of TMD pain [24], and in an eight-year cohort of people with pain-related TMD, baseline health-related quality of life and jaw functional limitation were associated with long-term pain intensity [25]. Onset, persistence, recurrence and recovery are therefore treated here as distinct transitions that may have partly distinct determinants.

### 4.4. What Role Plasticity Does and Does Not Yet Have Evidence for

That one factor may serve one or several of these roles has been stated in the literature for some time [9], but longitudinal demonstrations of a single, precisely defined variable occupying all three positions in the same individual are scarce. This warrants restraint. Role assignment should remain provisional and evidence-dependent, and repeated measurement is needed to separate a stable vulnerability from a change near onset and from a later maintenance influence.

The framework permits multiple positions; the data determine which assignments are justified in a given analysis.

### 4.5. Differential Response Is Already Measurable

Two OPPERA analyses show that comparable exposures need not have comparable effects. The association between perceived stress and incident TMD differed by COMT diplotype [18], and in a nested case–control study of incident painful TMD, baseline thermal-pressure and pinprick sensitivity measured by quantitative sensory testing modified the association between jaw injury and subsequent onset [26]. Neither study set out to measure adaptive capacity, and neither variable is renamed here. Their relevance is methodological: prespecified person-by-exposure interaction provides one empirical route for testing whether the impact of a predisposing, initiating, or perpetuating influence depends on independently measured characteristics of the individual.

## 5. Assigning Functional Positions Conservatively

The framework is useful only if positions are assigned reproducibly and withheld when the evidence does not support them. Table 2 sets out what kind of temporal evidence licenses predisposing, initiating, or perpetuating language. When timing is inadequate, the correct conclusion is that the position is temporally indeterminate; stating that uncertainty is preferable to completing the framework retrospectively.

A further distinction separates domain, functional position, and measurement. Bruxism, stress, sleep, trauma, and depression name constructs, not functions. Self-report, clinical signs, instrumented activity, retrospective history, repeated state measurement, and prospectively dated events are not interchangeable, and divergences between them are informative in their own right [12,27,28]. The framework therefore asks first what was measured and when, and only then what functional inference is available. Table 3 applies this principle across several domains.

## 6. Testable Predictions and Findings That Would Weaken the Framework

The framework is not intended to accommodate every possible finding. Its value depends on generating observations that could weaken, render unnecessary, or require revision of its components. The following predictions therefore state both the expected empirical pattern and the corresponding negative result that would challenge the proposal.

(1) Temporal position carries information beyond presence. In cohorts with sufficiently dense measurement, models that distinguish premorbid level, pre-onset change, and post-onset level should predict incidence and course better than models using presence alone. OPPERA has already shown that re-measurement at onset improves prediction relative to premorbid measurement [22]; the prediction is that this generalizes across datasets and variables. If temporal resolution reproducibly adds no information, the functional–temporal assignment adds little beyond factor presence.

(2) Associations for a given variable change with the temporal position at which it is analyzed. Baseline level, pre-onset change and post-onset level should not show equivalent associations for time-varying influences. If associations are invariant to temporal position across well-measured variables, role plasticity has little empirical content.

(3) At least some influences occupy more than one position. Repeated-measures studies should identify variables that behave as susceptibility markers in one temporal context and as onset-related or maintenance influences in another. If well-measured influences map to a single position regardless of timing, outcome, and context, the role-plasticity hypothesis is weakened.

(4) Prespecified person-by-exposure interactions replicate for at least some influences. If adequately powered, prespecified interaction analyses consistently fail to identify reproducible modification, the adaptive-capacity hypothesis adds little to an additive account. Conversely, isolated or post hoc interactions without temporal or biological plausibility would not, by themselves, support adaptive capacity.

(5) Onset, persistence and recovery have only partly shared determinants. If a single fixed set of variables with equivalent effects accounted for first onset, persistence, recurrence and recovery alike, distinguishing functional positions across the trajectory would be redundant.

(6) Persistent trajectories contain identifiable post-onset maintenance processes, while rapidly remitting episodes need not. If densely measured persistent episodes show no post-onset process associated with duration or recurrence beyond the initiating exposure, the perpetuating position requires re-specification.

The study design most directly suited to evaluating the framework would be an inception cohort beginning from a clearly defined TMD-free state, with repeated measurement at intervals short enough to resolve change around onset and with incident cases followed through remission, persistence, and recurrence. Such a design permits the same variable to be analyzed at several temporal positions without assigning its role in advance. Time-varying covariate models, multistate models, and joint models of repeated exposures and transitions are well suited to this task. The unit of analysis should be a specified phenotype and a specified transition—TMD-free to painful TMD, transient to persistent pain, remission to recurrence—and interaction analyses should be prespecified and adequately powered, since effect modification is harder to estimate reliably than main effects. Negative findings matter as much as positive ones: an influence that is common among patients but does not predict onset prospectively should not receive a predisposing or initiating label because it is clinically familiar, and an association measured after onset does not show that the same influence initiated the disorder.

## 7. Clinical Implications

The clinical contribution of the framework is not a new diagnostic category or treatment algorithm. Its intended innovation is a more disciplined way of assigning etiological meaning to information already collected in clinical practice. For an individual patient, the framework reduces to four questions: what made this person more susceptible; what changed or occurred around the beginning of this episode; what may be maintaining or reactivating the problem now; and what independently measurable characteristics of this person may increase or reduce the impact of those influences. The answers may change over time, one influence may appear in more than one answer, and some answers may legitimately remain temporally indeterminate.

### 7.1. Worked Clinical Example

Consider a hypothetical patient with no previous painful TMD who reports long-standing sleep disturbance and recurrent neck pain. After a period of increased psychosocial stress, the patient undergoes a prolonged dental procedure requiring sustained mouth opening and develops unilateral masticatory pain shortly afterward. During the following weeks, pain persists while frequent waking-state jaw clenching and pain-related worry are reported.

Applying the four questions together with Table 2 prevents these observations from being converted automatically into causes. The long-standing sleep disturbance and recurrent neck pain were present before onset and may be described as susceptibility characteristics, but their presence in this patient does not prove that they caused the episode. The prolonged mouth opening preceded symptom onset and is temporally compatible with a potential initiating influence; temporal proximity alone still does not establish causation. Jaw clenching and pain-related worry observed after onset are concurrent candidate maintenance processes. Unless longitudinal evidence links them to continued symptoms, recurrence, or slower remission, the preferred language remains “associated with” or “concurrent with” rather than “perpetuating”.

The fourth question concerns adaptive capacity. Independently measured characteristics such as pain modulation, coping-related variables, comorbidity, or other prespecified moderators may alter the impact of the exposures, but a favorable or unfavorable outcome cannot itself be used as evidence of high or low adaptive capacity. A conservative clinical formulation might therefore state that pre-existing susceptibility characteristics were present, that prolonged mouth opening temporally preceded onset and may have contributed to initiation, and that post-onset jaw clenching and pain-related worry are concurrent findings whose perpetuating role is not established. The purpose is to make uncertainty explicit rather than to retrospectively complete all three positions.

### 7.2. Communication with Patients

The following fire analogy is offered solely as a communication tool and should not be interpreted as evidence for, or as a mechanistic representation of, the framework. Dry wood does not produce fire, but it ignites more readily than iron, illustrating the predisposing position; a match must be struck, or another input must supply sufficient energy, illustrating the initiating position; and once ignition has occurred, continuing fuel and air influence whether the flame dies down or spreads, illustrating the perpetuating position. Dryness also shows why positions are not tied to objects, because it can make a material easier both to ignite and to keep burning. Two comparable structures exposed to the same match and fuel may still behave differently because moisture, fire-resistant materials, or suppression systems alter the consequences of a given exposure.

The clinical value afforded by this approach is limited in scope but specific in application. A patient can understand that having dry wood does not mean a fire must occur, that a match does not guarantee a sustained fire, and that two people exposed to apparently similar combinations may respond differently. This framing supports discussion of stress, oral behaviors, sleep, injury, anatomical characteristics, and systemic conditions as contributors with context-dependent roles rather than as single causes, which is closer to what the evidence supports than a checklist of alleged causes.

This framework pertains to etiological interpretation, clinical reasoning, and patient communication; it does not address treatment efficacy. Showing that an influence predicts onset or persistence does not show that modifying it prevents or resolves TMD, which remains a question for intervention studies.

## 8. Phenotype Specificity and Limitations

Most of the prospective evidence drawn on here concerns painful TMD, particularly examiner-classified myalgia and arthralgia in OPPERA and related cohorts. For this reason, the title and central framing of the revised manuscript explicitly focus on painful TMD. Structural and degenerative phenotypes have a substantially thinner prospective evidence base, and the framework should not be assumed to transfer unchanged to disc displacement, degenerative joint disease, subluxation, or joint noises. The distinction is conceptual as well as empirical: an influence may be predisposing for pain without being predisposing for an imaging outcome, an event may initiate symptoms without initiating degeneration, and modifiers of pain expression may differ from modifiers of structural change. Any extension of the framework should specify the phenotype and transition before functional positions are assigned.

The recent literature published within the past five years reinforces both the breadth of biopsychosocial associations and the continuing uncertainty around transition to chronic TMD pain: a 2024 systematic review identified multiple associated factors across studies using Diagnostic Criteria for Temporomandibular Disorders or Research Diagnostic Criteria for Temporomandibular Disorders, a 2022 critical review found limited evidence for predictors of acute-to-chronic TMD pain transition, and a 2026 population study associated painful TMD with several biopsychosocial characteristics [30,31,32]. These studies provide contemporary context but do not replace the temporally resolved prospective evidence needed to assign functional positions.

This article is subject to several limitations inherent to its conceptual design. It is not a systematic or scoping review, and the studies discussed were selected purposively because they illustrate, support, or constrain components of the proposed framework. This selection creates a risk that confirmatory or readily interpretable examples are overrepresented, while null, discordant, or less temporally resolved findings receive less emphasis. The cited studies should therefore not be regarded as an exhaustive or representative synthesis of the literature, and no formal risk-of-bias or certainty assessment was undertaken. Future systematic evaluations of the framework should explicitly incorporate null and discordant findings. The three-position structure is an organizing framework rather than an established requirement, and existing multifactorial accounts do not depend on it. The perpetuating position remains the most demanding component to define and may require re-specification if high-resolution longitudinal data show persistent episodes without a distinguishable post-onset maintenance process.

Two further constraints deserve emphasis. Adaptive capacity is only partly operationalized: effect-modification analysis is a defensible empirical entry point but does not exhaust what clinicians may mean by resistance, accommodation, or recovery. Moreover, existing longitudinal examples cluster in psychological, sensory, behavioral, and mechanical domains because those are the areas in which cohorts have achieved usable temporal resolution. The relative absence of social and contextual examples reflects limitations of the available evidence rather than evidence that those domains are unimportant. Finally, the fire analogy is a communication device only and carries no evidentiary or mechanistic claim.

## 9. Conclusions

Painful TMD has a multifactorial and biopsychosocial etiology, and the influences associated with its onset and course have been mapped across multiple domains. The present proposal separates an organizing framework from the hypotheses it generates. The framework asks whether an influence shaped susceptibility, contributed to the transition into clinical expression, or is associated with persistence, recurrence, or slower remission. The first hypothesis is that these roles must be assigned from temporal, contextual, and measurement evidence rather than treated as fixed properties of variables. The second is that independently measurable person-level characteristics may modify the impact of etiological influences, with prespecified effect-modification analysis providing one route for empirical testing.

Whether the framework is useful depends on evidence that does not yet exist in the required form. Densely measured inception cohorts could determine whether temporal position adds predictive information, whether the same influence occupies different positions when timing and outcome change, and whether prespecified person-by-exposure interactions replicate. Results that fail to support these predictions would weaken or require revision of the corresponding components. That possibility is a feature of the proposal rather than a limitation: the framework is intended to organize testable questions, not to make every observed association fit.

## Figures and Tables

**Table 2:** Evidence situations and the etiological-function language they support.

Evidence Situation	Inference Supported	Preferred Wording	What Should Not Be Claimed
Factor measured only concurrently with TMD	Temporally indeterminate	associated with; concurrent with	predisposing; initiating; perpetuating; causes
Characteristic present at baseline in a TMD-free cohort that predicts incidence	Predisposing characteristic/prospective predictor	prospectively predicted first onset	proven cause; initiating event, without further temporal evidence
Repeatedly measured exposure or state that changes before incident TMD	Potential initiating influence, if timing is sufficiently resolved	the change preceded and predicted onset	the factor is always initiating; inevitable cause
Discrete event dated before incident TMD and modeled as time-dependent	Initiating/precipitating	the event preceded and predicted incident TMD	the event inevitably causes TMD
Factor measured after onset that predicts continued symptoms, recurrence or slower remission	Perpetuating/prognostic	associated with persistence or recurrence; prognostic for continuation	cause of the original onset
Factor prospectively associated with remission or recovery	Recovery-associated/favorable prognostic	associated with recovery or remission	guarantees recovery
Interaction between a measured exposure and a person-level characteristic	Moderation/effect modification; evidence relevant to testing adaptive capacity	the effect of the exposure differed according to the characteristic	adaptive capacity itself was measured or established by the interaction alone
Same factor or domain supported at more than one temporal position	More than one functional position	acted as X in one temporal context and as Y in another	the factor permanently belongs to one position
Different measurement modalities give divergent associations	Measurement-specific inference	the specified self-report, clinical or instrumental measure predicted, or did not predict, the outcome	all modalities represent one interchangeable exposure

**Table 3:** Applications of the role-plasticity principle across domains.

Domain	Evidence Before Onset	Evidence Around Onset	Evidence After Onset	Interpretive Point
Psychosocial	Baseline psychological and somatic measures predicted first onset in OPPERA [11] and in independent cohorts [15,16].	Perceived stress measured repeatedly rose during the pre-onset period, and the association with incidence varied by COMT diplotype [18].	Catastrophizing and depression predicted progression of pain and disability in established TMD pain [20].	One domain, three different inferences; construct, timing and outcome decide which is available.
Jaw injury	Cross-sectional association with prevalent painful TMD, without temporal ordering [17].	Injury ascertained quarterly and modeled as time-dependent predicted incident painful TMD; most injuries were intrinsic [17].	Whether post-injury processes maintain the episode requires separate longitudinal evidence.	A dated event can support an initiating assignment; the word trauma alone cannot.
Experimental pain sensitivity	Some baseline sensory measures showed modest prospective associations with first onset [14].	Baseline thermal-pressure and pinprick sensitivity modified injury-related risk of onset [26].	Biopsychosocial characteristics, including sensory measures, change across TMD trajectories [23].	The same characteristic may be a predictor in one analysis and a moderator in another.
Sleep	Sleep disturbance was an independent predictor of first onset [13]; sleep-apnea symptoms predicted incidence [29].	These baseline measures do not resolve a change close to onset.	Concurrent association alone does not establish maintenance.	The framework follows the available temporal evidence instead of filling all three positions by default.
Oral behaviors and bruxism	Self-reported oral parafunctions predicted first onset, whereas examiner-assessed tooth-wear facets and joint noise did not [12].	Repeated measurement is required to establish whether change near onset contributes to initiation [22].	Outcome-specific effects on persistence require dedicated longitudinal evidence.	Self-report, clinical signs, instrumented recording and polysomnography are not interchangeable constructs [27,28].

## Data Availability

No new data were created or analyzed in this study. Data sharing is not applicable to this article.
